# Anti-inflammatory and immune modulatory properties of mesenchymal stromal cells

**DOI:** 10.1186/1479-5876-9-S2-I15

**Published:** 2011-11-23

**Authors:** Willem E Fibbe

**Affiliations:** 1 Dept. of Immunohematology and Blood Transfusion, Leiden University Medical Center, Leiden, The Netherlands

## 

Abstract not submitted for publication

